# Mixed method assesses Chinese rehabilitation students' knowledge, attitude, and practice of physical activity guidelines

**DOI:** 10.3389/fpubh.2026.1775288

**Published:** 2026-04-08

**Authors:** Manyue Luo, Wanqiang Chen, Qing Bai

**Affiliations:** 1 First Hospital of Lanzhou University, Lanzhou, China; 2 The University of Edinburgh, Edinburgh, United Kingdom

**Keywords:** clinical education, physical activity, rehabilitation students, rehabilitation therapy education in China, undergraduate education

## Abstract

**Background:**

Physical inactivity is a major global health risk. Physical therapists, as key health promoters, are well-positioned to counsel patients on physical activity (PA) using evidence-based guidelines. However, studies globally indicate physiotherapists' knowledge of PA guidelines (e.g., WHO recommendations) is often insufficient. In China, the state of PA guideline education and knowledge among rehabilitation students—future physiotherapists—remains unexplored.

**Objectives:**

This mixed-methods study aimed to: (1) assess the level of teaching and knowledge of PA guidelines among Chinese rehabilitation students, and (2) discuss ways to better incorporate PA guideline teaching at the undergraduate level.

**Methods:**

An explanatory sequential design was employed. Phase 1 involved an online questionnaire distributed to 105 recent graduates (post-2020) from Chinese rehabilitation programs, assessing their knowledge, educational exposure, and self-reported clinical practices related to PA guidelines. Phase 2 consisted of 10 online, semi-structured interviews with experienced rehabilitation therapists to gain in-depth insights into guideline application and promotion in practice. Quantitative data were analyzed using descriptive statistics, chi-square tests, and ordinal logistic regression. Qualitative data were analyzed thematically.

**Results:**

Quantitative findings revealed that 66.7% of surveyed students reported not being taught PA guidelines during their undergraduate studies. Only 10.4% could correctly answer all three core knowledge questions about the WHO PA guidelines. A significant association was found between learning PA guidelines at university and a higher self-reported frequency of initiating discussions about physical inactivity with patients during internships [adjusted OR = 4.73, 95% CI (2.10, 10.67), *p* < 0.001]. Interview data identified three main themes: (1) the recognized benefits of appropriate PA for patient recovery and adherence, (2) the current inadequacy of PA guideline teaching in undergraduate curricula, with a strong recommendation for integrating theory with clinical practice, and (3) the need for selective promotion of PA guidelines in clinical settings, with outpatient departments highlighted as particularly suitable contexts.

**Conclusion:**

There is a significant gap in PA guideline education and knowledge among rehabilitation students in China. Integrating PA guideline instruction into undergraduate curricula, particularly in conjunction with clinical practice, is crucial. Furthermore, promoting PA guidelines may be most feasible and effective in outpatient clinical settings.

## Introduction

Lack of physical activity (PA) is the fourth leading risk factor for deaths related to non-communicable diseases (NCDs), accounting for approximately 6%−10% of NCD mortality globally (1). However, about 17.7% of the global population aged 15 years and above engage in no form of PA, and nearly 58% fail to meet the World Health Organization's recommendation of at least 150 min of moderate-intensity activity per week (2, 3). Individuals with insufficient PA are estimated to use more healthcare services than their active counterparts, thereby imposing a substantial cost burden on public healthcare systems (4). If the current levels of physical inactivity remain unchanged, it is projected to result in direct healthcare costs of approximately US$520 billion by 2030, creating a heavy strain on public health (5).

To address this challenge, the World Health Organization released its latest Guidelines on Physical Activity and Sedentary Behavior in 2020, providing evidence-based recommendations on the type, duration, and intensity of PA for different populations, along with specific guidance on reducing sedentary behavior to promote public health (6). However, effectively promoting PA to the public remains challenging, partly because information about the beneficial types, duration, and intensity of PA is still insufficient (7). This key information is essential for clinical healthcare professionals, as it enables them to design and promote personalized PA prescriptions (8).

Against this backdrop, rehabilitation medical professionals, particularly physical therapists, play an indispensable role. PA constitutes a core component of rehabilitation medicine (9). Patients receiving care from physical therapists often present with physical functional impairments resulting from illness, and a central responsibility of the therapist is to restore patient function through the development of individualized short- and long-term physical activity prescriptions (10). Consequently, PA is not only critical for rehabilitation therapy but also serves as the foundation for individuals to maintain basic activities of daily living (11, 12). Compared to other healthcare professionals, patients are more likely to seek PA advice from physical therapists during brief clinical encounters, owing to their recognized expertise in health promotion (12). These brief clinical interactions provide valuable opportunities to address health issues stemming from physical inactivity (13). Furthermore, given the typically extended duration of rehabilitation care, the sustained therapeutic relationships physical therapists establish with patients increase the likelihood that patients will rely on and apply the latest PA guidelines to maintain appropriate activity levels (14).

Research indicates that despite physiotherapists' limited knowledge of PA, they are still making efforts to promote it (15, 16). This precisely underscores that, through systematic education and training to equip physical therapists with accurate and comprehensive knowledge of guidelines, they have significant potential to serve as ideal advocates and implementers of PA across the domains of prevention, promotion, and rehabilitation (17). Therefore, clarifying the professional responsibilities of physical therapists and strengthening their education on PA guidelines constitutes a critical link in effectively integrating public health goals, clinical practice, and long-term patient health management.

However, translating this potential into effective clinical practice first requires addressing the significant knowledge gap that physical therapists themselves have regarding physical activity guidelines. The main findings from studies examining physiotherapists' knowledge of PA guidelines are summarized in Table 1. Multiple cross-sectional studies from different countries (e.g., the United Kingdom, Israel, India, and Nigeria) reveal a common issue: physiotherapists' grasp of the recommended dose, intensity, and types of physical activity issued by authoritative bodies such as the World Health Organization is suboptimal, and their level of knowledge falls far short of the standard required to effectively guide patients.

**Table 1:** Summary of studies (*n* = 4) exploring knowledge of PA guidelines among physiotherapists.

References	Country & design	Participants	Finding
Lowe et al. (13)	United Kingdom A cross-sectional survey	463 individuals (89%) were qualified physiotherapists. 51 individuals (10%) were student physiotherapists. 8 individuals (1.5%) were support workers.	Only 16% of the 522 participants could accurately answer the three questions related to PA guidelines.
Yona et al. (18)	Israel Cross-sectional online survey	1,062 (75%) Israeli physiotherapists	Only 6.8% of physiotherapists were aware of the recommended level of PA for adults by WHO, and only 4% knew the recommendations for adolescents.
Aditya Jadhav et al. (19)	India Cross-sectional online survey	185 Indian professional physiotherapists	Only 19% of physiotherapists were able to give correct answers in all three areas of the WHO PA Guidelines.
Oyeyemi et al. (10)	Nigeria PA survey questionnaire	153 physicians and 94 physiotherapists	More than 40% of physiotherapists and doctors were unaware of the correct dose of PA that could provide health benefits to their patients. Physicians had better overall attitudes toward promoting PA than physiotherapists (*p* = 0.048).

A closer analysis of these studies reveals several key patterns. First, physiotherapists generally have a vague awareness of the specific activity recommendations for adults, such as 150 min of moderate-intensity activity per week, with only a small proportion (ranging from 6.8% to 19% across studies) able to answer related questions accurately. Second, even among those with overall awareness of the guidelines, the ability to translate them precisely into personalized “exercise prescriptions” suitable for individual patients remains insufficient. Furthermore, these knowledge gaps are prevalent across countries with varying levels of development and healthcare systems, suggesting a potential widespread lack of systematic integration and emphasis on the latest, specific content of physical activity guidelines in global physiotherapy education curricula.

This situation constitutes a fundamental contradiction: while physiotherapists are expected to play a central role in health promotion, the key knowledge base on which they rely for practice is notably deficient. Therefore, systematically evaluating and strengthening physiotherapists' knowledge in this area is not only an urgent priority for improving their clinical competence but also a necessary precondition for realizing their public health expectation as “ideal advocates” for PA.

In China, the training of rehabilitation therapists typically takes place at the undergraduate level, where rehabilitation students obtain their professional qualifications through university courses and choose to specialize in fields such as Physical Therapy (PT), Occupational Therapy (OT), or Speech Therapy (ST) for their future careers (20, 21). As a result, most professional physiotherapists in China are students who have studied rehabilitation-related disciplines during their undergraduate education (22). China's rehabilitation-related majors are mainly Rehabilitation Therapy and Sports Rehabilitation (23). As of 2020, more than 150 undergraduate colleges and universities across the country have opened Rehabilitation Therapy majors, and more than 50 colleges and universities have opened Sports Rehabilitation majors, with a total of about 10,000 undergraduate rehabilitation majors graduating each year (24). However, the present stage of undergraduate rehabilitation education in China primarily emphasizes equipping students with the necessary rehabilitation treatment techniques, and preventive knowledge, such as the correct dosage of PA and the science of physical health, may receive limited attention (23, 25). This suggests that there might not be sufficient emphasis on and instruction regarding PA Guidelines in the education of rehabilitation student curriculum at the undergraduate level. Additionally, the researcher has noted that there is currently no specific research data available on the knowledge of PA Guidelines among Chinese rehabilitation students.

Most of the previous related research has used cross-sectional online surveys to assess physiotherapists' understanding of PA Guidelines by assessing responses to three questions (13, 18, 19, 26). However, little research has focused on the teaching of PA Guidelines during the training and education of physiotherapists. Oyeyemi et al. (10) employed offline questionnaires, which facilitated direct interactions between researchers and participants, resulting in a precise comprehension of questions about PA information and yielding a complete response rate of 100%. However, due to the limitations of offline questionnaires, the study in Nigeria was limited to five recruited hospitals and may not be representative of the wider population of physiotherapists. Additionally, all forms of questionnaire surveys can only reflect physiotherapists' understanding of PA Guidelines based on self-report and cannot explore the extent of implementing the guidelines in practice (19, 27). Understanding how to promote PA Guidelines in the clinical healthcare environment requires the use of different research methods for investigation. Therefore, this study adopted a mixed-method approach, using both online questionnaires to assess knowledge and online interviews to explore practical promotion in medical settings. By collecting responses from recent years' graduates who completed rehabilitation courses at various universities and graduated after the year 2020, the aim was to obtain specific data on the education and understanding level of PA Guidelines among recent Chinese rehabilitation graduates. Additionally, through interviews with experienced professional rehabilitation therapists, the study explored the practice of promoting PA in clinical healthcare settings.

### Research aim

The purpose of this study was to explore the extent to which Chinese rehabilitation students are aware of the PA Guidelines and how the PA guideline can be better promoted in practice settings. The specific objectives were:

1. Data on the teaching and understanding of PA Guidelines among Chinese rehabilitation students.

2. Way to better incorporate the teaching of PA Guidelines at the undergraduate level.

3. Suggestions to help better promote PA Guidelines in the clinical healthcare setting.

## Method

### Ethics

Ethical clearance was obtained from the University of Edinburgh, Moray House School of Education and Sport Research Committee before the commencement of the study (Ref: ISPEH2023-S2421795).

### Design

A mixed-methods explanatory sequential design was adopted to investigate Chinese students' knowledge of PA guidelines and related clinical practice. This design is characterized by a two-phase approach: in the first phase, quantitative survey data were collected to examine broad patterns and prevalence; in the second phase, qualitative interviews were conducted to explain, contextualize, and elaborate on the quantitative findings obtained earlier (54). Under this framework, the study first administered and analyzed a quantitative questionnaire regarding rehabilitation students' knowledge and practice of physical activity guidelines. Based on the quantitative results—particularly points of contradiction or complex patterns requiring deeper interpretation—the subsequent qualitative interview study was purposively designed. A key strength of this approach is that the qualitative data collection was deliberately targeted to help explain the “why” and “how” behind the quantitative outcomes, thereby providing richer insight and methodological triangulation.

### Online questionnaire

#### Participants

Participants completed a rehabilitation-related undergraduate degree in China and graduated after 2020.

#### Recruitment

The main recruitment was carried out via social media groups on the widely used Chinese technology application, WeChat functions as a versatile social media platform and is extensively utilized on mobile devices and other portable gadgets (28). Most colleges and universities will use WeChat to deliver messages and send notifications to students at that school (29). Additionally, recruitment activities were conducted through email, specifically at the researcher's undergraduate institution.

The online data collection commenced on June 4, 2023, and the final response was received on July 7, 2023.

#### Procedure

The researchers used Qualtrics to design a bilingual (Chinese and English) online questionnaire, which was specifically developed for this study. The questionnaire comprised a total of 20 sections, with a focus on rehabilitation students' knowledge, education, and practices regarding the promotion of PA. All questions were closed-ended or multiple-choice to avoid self-bias. Some questions provided multiple answer choices.

To ensure both linguistic equivalence and cultural appropriateness, a systematic translation and adaptation process was conducted following the guidelines for cross-cultural adaptation proposed by Beaton et al. (30). Initially, forward translation and synthesis were carried out: two bilingual researchers specializing in rehabilitation independently translated the original English questionnaire into Chinese. They then collaborated with a senior rehabilitation therapist to discuss and reconcile the two translated versions, resulting in a preliminary Chinese draft. Special emphasis was placed on ensuring the accuracy of professional terminology and contextual localization for clinical practice.

Finally, a pre-test was administered to 10 rehabilitation therapists who met the formal inclusion criteria of the study. Using the “Think-aloud Technique” (31), participants were asked to verbalize their understanding of each question, identify potential ambiguities, and describe their thought processes while completing the questionnaire. Based on the feedback received, the wording of certain items was refined to improve clarity and cultural appropriateness before the final version of the questionnaire was officially released.

#### Measurement

The core section of the survey comprised 16 items. The complete questionnaire is included in Appendix 1. The instrument is structured into the following three domains.

1. Demographic and screening section

Six items (Items 1–6) were administered to screen participants and collect demographic data. These items pertained to institutional affiliation, academic degree, year of graduation, current professional or internship setting, and relevant prior experience. This screening ensured alignment with the study's predefined inclusion criteria.

2. Knowledge assessment module

Knowledge was evaluated using five items (Items 7–11). Two items employed a dichotomous (yes/no) response format to ascertain whether formal education on physical activity was received during undergraduate training. The remaining three items were adapted from Lowe et al. (13) to gauge respondents' knowledge of the World Health Organization's physical activity guidelines, specifically the recommended levels of moderate- and vigorous-intensity activity for adults. The validity of these items is supported by their prior use and validation in a large-scale UK study (*N* = 522).

3. Attitudes and clinical practices scale

Attitudes and self-reported practices were assessed via four items (Items 12–15), adapted from the instrument developed by Aditya Jadhav et al. (19) for evaluating rehabilitation students' attitudes toward physical activity and its clinical promotion. The items measured the following constructs: initiating conversations about physical inactivity, formally assessing a patient's physical activity status (using screening tools), delivering brief interventions for physical inactivity when clinically indicated, and supervising physical activity engagement outside of formal therapy sessions. Responses were recorded on a 4-point Likert scale (1 = Never, 2 = Sometimes, 3 = Usually, 4 = Always). The internal consistency reliability for this 4-item scale was calculated, yielding a Cronbach's alpha coefficient of 0.721, which indicates an acceptable level of reliability for the questionnaire.

### Data analysis

To measure knowledge levels, the researcher employed the approach of Aditya Jadhav et al. (19), considering responses to questions (Questions 9, 10, 11) related to the WHO's PA Guidelines. The correct answers to individual questions and all three questions were evaluated among 105 participants. To explore whether learning the PA Guidelines during undergraduate studies had any impact on subsequent clinical practice, researchers initially employed chi square tests to analyze the association between Chinese rehabilitation students who had learned the PA Guidelines (33.3% of the sample, *n* = 35) and those who had not (66.7%, *n* = 70) regarding two outcomes: the frequency of discussing physical inactivity and the frequency of encouraging rehabilitation clients to engage in PA outside of routine therapy (categorized as Never/Sometimes/Usually/Always). To further control for potential confounders, two separate multivariable ordinal logistic regression models were fitted. The dependent variables were the ordinal frequencies as described. The key independent variables were awareness during university studies (for the conversation model) and awareness during internship (for the supervision model). Both models were adjusted for academic degree, healthcare institution type, and years of professional experience. All responses were verified, compiled in Microsoft Excel, and then imported into IBM SPSS Statistics 27 software for analysis.

### Online interviews

#### Participants

Ten professional rehabilitation therapists with at least 1 year of internship or work experience in rehabilitation healthcare institutions were interviewed. In semi-structured interview studies, if the respondent fails to provide new information, this may suggest that the sample size is sufficient and representative (15). There has been a successful semi-structured interview study conducted with 10 teachers to obtain their experiences in practical teaching (32). This study also included 10 interviewees, all experienced professional therapists, to gain insight into their recommendations for teaching and applying the PA guidelines in their practice. They met the following criteria: (1) aged over 20 years, (2) certified professional rehabilitation therapists with at least 1 year of internship, or work experience in rehabilitation healthcare institutions, (3) proficient in either Chinese or English.

#### Recruitment

Participants were invited online by the researcher through email and were all located in China. Participants had a mean age of 23.7 (standard deviation (SD) = 1.9) and the average internship and work experience duration was 1.7 years, SD = 1.61). Most participants were female (*n* = 9), and to protect their privacy, each participant was assigned a month code based on the interview sequence.

#### Procedure

All participants provided informed consent before the interview. The interviews were conducted and recorded using Microsoft Teams.

#### Measures

A semi-structured interview schedule specifically designed for this study was used to collect data. The semi-structured approach aims to provide flexibility, allowing interviewers to explore participants' thought processes, address potential ambiguities, and enable researchers to verify the meaning of participants' answers (33).

The study focused on how to teach and promote PA Guidelines in the clinical healthcare environment. The interview questions were neutrally framed rather than leading and underwent review by a professional physiotherapist to ensure clarity. The interviews first explored the connection between PA and physical therapy, followed by discussions on the teaching and practical significance of PA Guidelines in the current rehabilitation therapist education. In the last five questions (Questions 7 to 11), the researchers referenced the questions used in the questionnaire design by Aditya Jadhav et al. (19) and Oyeyemi et al. (10), as well as guidance for future research directions, to investigate more deeply into the application and promotion of PA Guidelines in the clinical healthcare setting. The detailed semi-structured interview design is attached as Appendix 2.

### Data analysis

**Figure 1:**
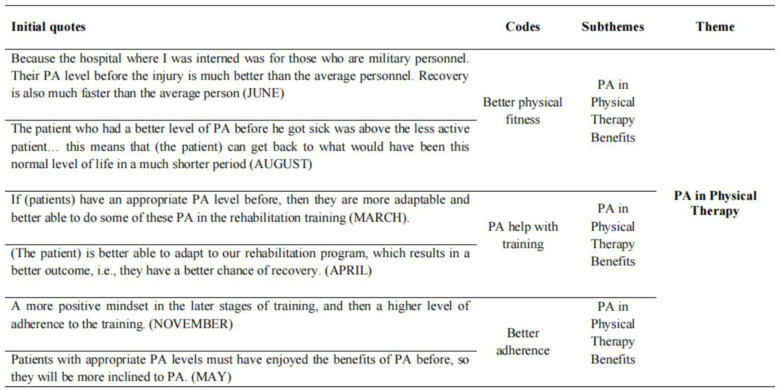
Illustrative analysis example, indicating the pathway from initial quotes to the theme of the interview.

After professionally transcribing the interview data, accuracy checks were conducted. The researchers used thematic analysis, one of the widely used qualitative analysis methods in social science (34), to identify key patterns in the data. To guide this process, the researchers followed the six-phase thematic analysis framework proposed by Braun and Clarke (35). Firstly, the researcher was familiarized with the interview through repeated readings of the transcripts, using the interview design questions as initial codes and generating preliminary codes with the qualitative data analysis software NVivo 14, which was associated with interesting features in the interview. Next, additional themes were identified and reviewed these themes to ensure consistency with the initial codes. Subsequently, the researcher defined and named these themes and connected the research findings with relevant literature (35) to deepen understanding and interpretation of these themes. Figure 1 shows an example that provides a snapshot of analysis to illustrate this process more clearly.

## Results

### Quantitative data

#### Demographics

The survey was randomly distributed among approximately 418 graduates of rehabilitation-related degrees and received 112 responses. The response rate was 26.8%. Seven questionnaires (*n* = 7) were excluded as they were implausible. The valid sample size for this study was 105. Given that the estimated population of rehabilitation students in China is approximately 10,000, the sample represents about 1% of the total population (24). In social science and medical survey research, a sample of this size is generally considered reasonable and acceptable. Moreover, all expected cell frequencies in the chi-square tests exceeded 5, supporting the reliability of the statistical results. *Post-hoc* power analyses were conducted to further assess the adequacy of the sample size for key analyses.

For the analysis examining the association between whether PA guideline education was received during university studies and whether physical inactivity was actively discussed with rehabilitation clients during internships, the observed statistical power was 0.96 (effect size *w* = 0.443, α = 0.05, *N* = 105). For the analysis of the relationship between whether PA guidelines were heard of during internships or work and whether patients were supervised to engage in PA outside of therapy, the observed power was 0.87 (effect size *w* = 0.36, α = 0.05). These *post-hoc* power estimates, both well above the conventional threshold of 0.80, indicate that the sample size was generally adequate to detect the observed effects with sufficient statistical power.

**Figure 2:**
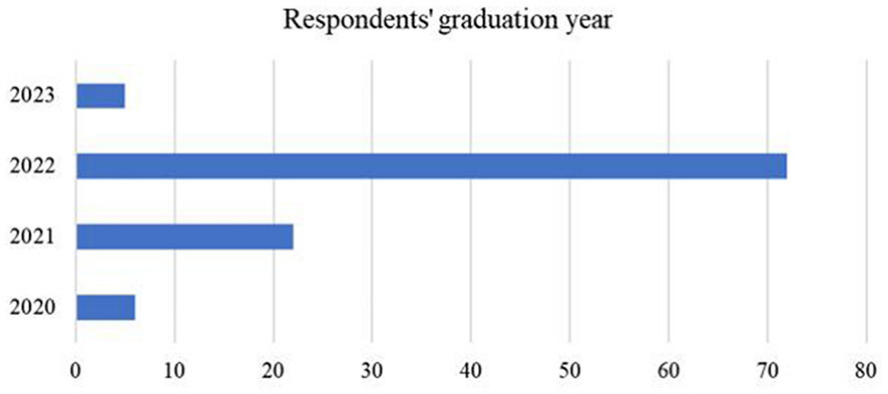
Reported different graduation years of rehabilitation students in online questionnaire. Vertical numbers represent the number of respondents who graduated in that year.

**Figure 3:**
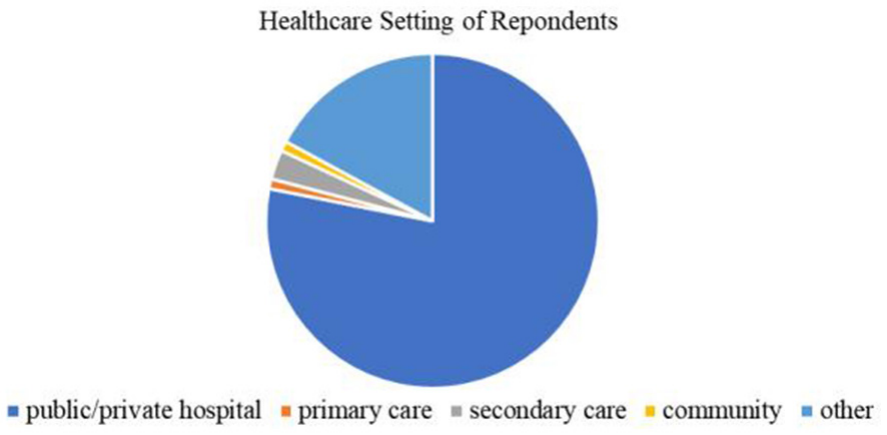
Reported different internships or work in clinical healthcare settings by rehabilitation students in online questionnaire.

Eighty percentage of the sample (*n* = 84) were in major rehabilitation therapy, 18.1% were in sports rehabilitation, and 1.90% (*n* = 2) were in other rehabilitation-related majors. Participants all reported different graduation years (Figure 2). Most participants (78.1%, *n* = 82) had work or internship experience in public or private hospitals, and all participants had internship or work experience in a variety of healthcare settings (Figure 3). The survey received feedback from 28 different universities in China. The list of the schools collected, and their corresponding numbers is in Appendix 3.

#### Teaching and understanding of PA guidelines

Across the 28 Chinese universities surveyed, 66.7% (*n* = 70) of the participants explicitly stated that they had not been taught about PA Guidelines during their undergraduate studies, and their knowledge of the three aspects of PA Guidelines detailed in Table 2, with only 10.4% (*n* = 11) of the respondents answering all three questions correctly.

**Table 2:** Statistical rehabilitation students' correct answers to three questions in PA guidelines.

PA guideline questions	Number of correct responses	Percentage of respondents who answered correctly (%)
Q: How many minutes of moderate-intensity PA is recommended per week for adults? A: 150	30	28
Q: How many minutes of vigorous-intensity PA is recommended per week for adults? A: 75	21	20
Q: On how many days per week is it recommended that adults undertake strength training? A: 2	17	16

#### Application of PA guidelines in practice

57.14% (*n* = 60) of the participants had not heard of PA Guidelines in internship or work, participants were asked to estimate the frequency with which they carried out some specific actions related to the promotion of PA in healthcare settings, the results of which are presented in Table 3.

**Table 3:** Frequency of respondents formally assess PA status and providing brief intervention.

	Never		Sometimes		Usually		Always	
Question	(Count)	(%)	(Count)	(%)	(Count)	(%)	(Count)	(%)
Formally assess PA status (use screening tools)	15	14.3	46	43.8	27	25.7	17	16.2
Provide brief interventions for physical inactivity when required	6	5.7	39	37.1	40	38.1	20	19.1

A significant association was found between learning PA guidelines at university and the frequency of initiating discussions about physical inactivity (χ^2^ *p* < 0.001; Cramer's *V* = 0.443, moderate strength). Similarly, awareness of guidelines during internship was significantly associated with the frequency of encouraging out-of-therapy PA (χ^2^ *p* < 0.01; Cramer's *V* = 0.360, moderate strength). For the ordinal regression model adjusted for covariates, learning PA guidelines during university was significantly associated with a higher frequency of initiating conversations [cumulative OR= 4.73, 95% CI (2.10, 10.67), *p* < 0.001]. This indicates that exposure to the guidelines was associated with 4.73 times greater odds of being in a higher frequency category. None of the covariates were significant in this model: academic degree (OR = 0.48, *p* = 0.110), institution type (OR = 1.21, *p* = 0.671), and experience (OR = 0.76, *p* = 0.527). For the outcome of supervising PA outside therapy, guideline awareness during internship was not statistically significant after adjustment (OR = 1.83, 95% CI: 0.88–3.81, *p* = 0.105). Similarly, none of the covariates showed significant effects: academic degree (OR = 0.76, *p* = 0.537), institution type (OR = 1.37, *p* = 0.480), and experience (OR = 1.07, *p* = 0.879).

### Qualitative data

**Figure 4:**
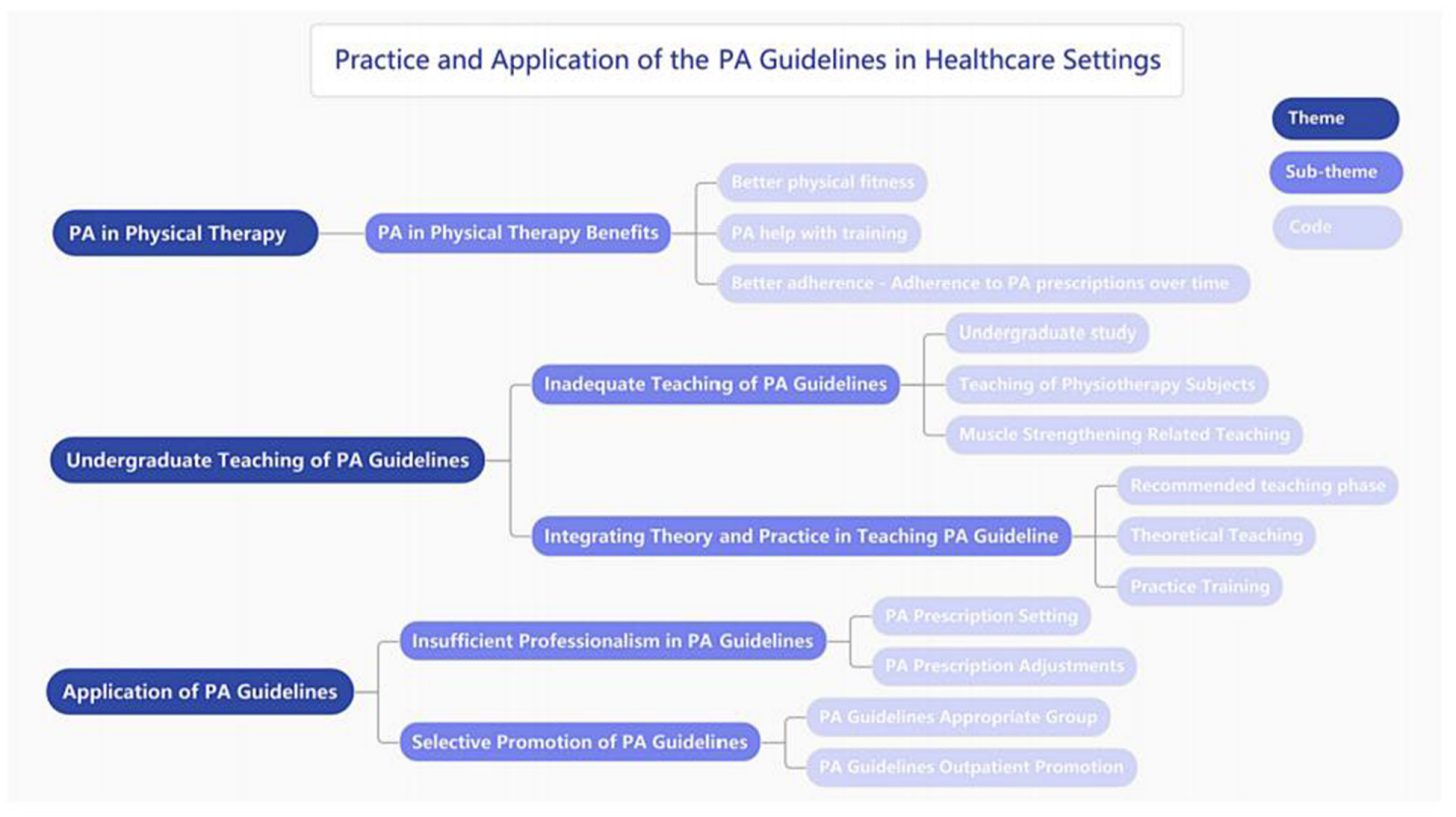
Qualitative data themes, subthemes, and codes in the interview.

Interviewees discussed the practice and application of the PA Guidelines in healthcare settings and made suggestions on how to teach and help promote the PA Guidelines. From interview data, the researcher identified three themes: PA in physical therapy, undergraduate teaching of PA Guidelines, and application of PA Guidelines, each of which contained several subthemes (see Figure 4).

#### Theme 1: PA in physical therapy

Participants demonstrated that an appropriate PA level can help rehabilitate patients lead to a better prognosis and improve their chances of successful rehabilitation.

#### Subtheme 1: PA in physical therapy benefits

All 10 interviewees agreed with the idea that, among patients with similar levels of dysfunction, those who had an appropriate level of PA before the disease had a better chance of recovery than those who were less active, and the reasons given by the interviewees can be summarized as follows:

1. Better physical fitness

According to interviewees' experiences, PA is described as contributing significantly to the prognosis of rehabilitative illnesses:

Because the hospital where I was interned was for those who are military personnel. Their PA level before the injury is more appropriate than the average. Recovery is also faster (June).

(The patient who had an appropriate level of PA) can get back to a normal level of life in a much shorter period (August).

2. PA help with training

An appropriate PA level can help the patient perform movements better during training:

If (patients) have an appropriate PA level before, then they are more adaptable and able to do some PA in the rehabilitation training (March).

(The patient with an appropriate PA level) is better to adapt to our rehabilitation plan (PA prescription), which results in a better outcome, i.e., they have a better chance of recovery (April).

3. Better adherence—Adherence to PA prescriptions over time

Adherence means that the patient can follow the treatment prescribed by therapists. Rehabilitation is a long-term treatment that takes a long time to adhere to, and an appropriate PA level can help the patient “have a more positive mindset in training, a higher level of adherence to the training” (November). This is mainly because “(patients with appropriate PA levels) must have enjoyed the benefits of PA before, so they will be more inclined to PA” (May). As a result, “they are less tolerant of lying in bed than non-exercisers, and they are more eager to get back to health, and they tend to ask the therapists how they can do extra PA after the treatment” (June).

#### Theme 2: undergraduate teaching of PA guidelines

All 10 interviewees mentioned that the curriculum of their undergraduate physiotherapy may be more biased toward physiotherapy techniques such as focusing on mastering the functional anatomy of muscles and teaching movements that strengthen a particular muscle.

##### Subtheme 1: inadequate teaching of PA guidelines

One participant mentioned that she was not able to answer questions related to a PA prescription when consulted: “But actually when I'm in a hospital like I meet a patient and then he asks me,” “how many times a week do you think it would be better for me to PA now,” or “how many sets of PA would be better for me to PA” or “how long would it be better for me to have PA.” But I can't answer those questions. But when I read this PA Guideline, I feel like I would know something about these questions that he asked, and I can tell him again' (February).

##### Subtheme 2: integrating theory and practice in teaching PA guideline

All 10 interviewees suggested a combination of theory and practice for the inclusion of PA Guidelines at the undergraduate level. They gave similar reasons for this, with the participant (September) representing that “it would be better to include it in the teaching of the (PA Guideline) both in basics and the internship, understanding the basics first and then applying them better in practice.” Interviewees particularly emphasized the significance of teaching in practice “to make students understand the specific application of PA Guidelines when it comes to clinical…and the relationship between setting the PA prescription and the recommendations from PA Guidelines, to enable teaching and learning to be integrated” (June).

##### Theme 3: application of PA guidelines

Rehabilitation therapists made the critical point that PA Guidelines may not be fully applicable to complex clinical healthcare situations, and therefore there is a need to be selective in the promotion of PA Guidelines.

###### Subtheme 1: insufficient professionalism in PA guidelines

Interviewees suggested that PA Guidelines may not be fully applicable to complex healthcare settings. When 10 professional rehabilitation therapists were asked whether muscle strengthening could be achieved up to three times per week (the PA Guidelines recommend three times per week for patients with long-term disease), the researcher get answers that in actual medical practice, for patients requiring muscle strengthening, the PA prescription would be significantly more than three times per week. In addition, the progress of the disease affects the PA prescription developed by the therapist. “A patient in the early or late stages of the disease may not have the same amount of PA prescription. Especially for mid-stage and early-stage patients, they may not be particularly stable, so the amount of training will be relatively lower for them” (November).

###### Subtheme 2: Selective promotion of PA guidelines

Interviewees suggested that the promotion of PA Guidelines needs to consider the appropriateness and specialization of different groups and not the whole healthcare environment is suitable for the promotion of PA Guidelines, for example, “patients in the ICU (Intensive Care Unit) where the most important thing is to keep them alive, everything else may have to be done afterward” (April). In terms of promoting the PA Guidelines, the interviewee suggested that “think about promoting it from a more appropriate group” (April). Another interviewee indicated that “it could be prioritized to promote the PA Guidelines in outpatients… because they may be more receptive, safer, and effective” (February).

## Discussion

This study examined the current status of knowledge, education, and practice regarding Physical Activity (PA) guidelines among rehabilitation therapy students in China. Survey data from students across 28 universities revealed that most had not received systematic instruction on PA guidelines during their undergraduate studies, which was associated with a limited understanding of the core content of the guidelines. Further analysis indicated a significant association between whether students had systematically learned PA guidelines and their reported future frequency of promoting PA in clinical practice.

To assess the strength of the associations between variables, an effect size analysis was performed. The results indicated a significant positive association between students'? exposure to PA guidelines during university education and their self-reported frequency of initiating PA-related discussions with patients during internship or early practice (Cramer's *V* = 0.443, reflecting a medium effect size). After adjusting for academic degree, type of healthcare institution, and years of experience, guideline learning during university remained a significant factor associated with conversation frequency (OR = 4.73). Based on the current sample (*N* = 105), students who had learned the guidelines had approximately 4.73 times higher cumulative odds of reporting a higher frequency of such conversations than those who had not. This cross-sectional association suggests that recalling exposure to PA guidelines during training is strongly associated with self-reported communication behaviors. Furthermore, awareness of PA guidelines during internships was also significantly associated with self-reported encouragement of patients to engage in PA outside treatment sessions (Cramer's *V* = 0.360, reflecting a medium effect size). However, in a regression model that adjusted for the same covariates, the relationship between guideline awareness and supervision behavior was attenuated and did not reach statistical significance (OR = 1.83, *p* = 0.105). The differing patterns of association between the university education phase and the internship phase warrant further investigation. Despite these variations, PA guideline education during internships may still show some association with the development of supervisory behaviors. Although the adjusted model did not confirm a statistically significant independent effect, the unadjusted association (Cramer's *V* = 0.360) and the direction of the odds ratio (OR > 1) suggest a potential trend that could be explored in larger samples.

Separately, the qualitative interviews offered a complementary, context-rich perspective that helped interpret the divergent quantitative findings. Participants consistently stressed the importance of integrating PA guidelines into professional education curricula. A shared view emerged that university education establishes an essential foundational knowledge framework, underscoring the potential critical role of PA education during undergraduate training. In parallel, the internship phase was characterized as a period of applied skill development, yet one often constrained by intense time pressure and competing clinical priorities. Notably, physiotherapists practicing in outpatient settings highlighted that therapist-led promotion of PA appeared more feasible in that context, given the workflow and patient interaction patterns. This observation provides a preliminary, hypothesis-generating insight for future studies examining how clinical context may shape the effectiveness of PA guideline education.

Methodologically, this study employed an explanatory sequential mixed-methods design. This approach enabled a transition from identifying a macro issue—the theory-practice gap—to examining contextual details. Quantitative results emphasized the broad importance of formal education, while qualitative interviews provided frontline perspectives: physiotherapists viewed outpatient settings (with higher patient volume and direct interaction) as more suitable for brief PA guidance than other clinical environments. Participants widely noted that university education built a foundational “knowledge framework,” and internships offered key opportunities to apply theory in practice. However, they also reported that internships, while valuable for exposure, were often limited by time pressures and competing clinical priorities, hindering the practical use of guideline knowledge. In summary, undergraduate education appears essential for fostering proactive communication, whereas structured support during clinical practice may help bridge the gap between knowledge awareness and behavioral implementation. These exploratory insights, based on a limited sample, are intended to generate hypotheses for future research on optimizing rehabilitation education, rather than to prescribe specific curriculum changes.

### Comparison of literature and plausible explanations

#### Teaching PA guidelines to Chinese rehabilitation students

The results of this study are consistent with the predictions of previous related research (7). Currently, Chinese rehabilitation undergraduate students do not receive sufficient teaching of PA Guidelines. To some degree explaining why most professional physiotherapists do not have an appropriate understanding of the PA Guidelines. Notably, promoting PA in the absence of adequate knowledge is not advisable (36) and the PA prescription in PA Guidelines is very practical in healthcare settings, it can somehow complement the expertise of physiotherapists (37). In addition, the chi-square test results suggest that PA Guidelines instruction at the undergraduate level may influence physiotherapists' subsequent promotion of PA guidelines in hospitals. This finding aligns with a survey conducted among American physical therapy students, which discovered that these students possess a comprehensive understanding of PA Guidelines and value their role in promoting PA (36). Therefore, incorporating the PA Guidelines curriculum is crucial for Chinese rehabilitation students.

#### Health benefits of PA for rehabilitation

During the interview phase, 10 respondents consistently reported on the prognostic benefits of appropriate PA levels before disease onset for rehabilitation based on their clinical experience and observations. This result is similar to the findings of Garza (38), appropriate levels of PA had a positive prognostic effect on cardiac rehabilitation (39). Pilot trials have shown that PA prescriptions by physiotherapists can help chronic pain patients maintain an appropriate PA level, and PA also has a positive impact on patients' physical health (14, 40). As a key component of therapeutic intervention, it's almost certain that PA plays a central role in promoting the health of physiotherapy patients.

#### PA promotion in clinical healthcare settings

During the interviews participants mentioned that as a public guideline, PA Guidelines may not cover all different diseases' PA prescriptions in the medical context. The occurrence and development of diseases may require physiotherapists to personalize and adjust PA prescriptions at different stages (41, 42). For patients who need long-term treatment, it is essential to trust the expertise of physiotherapists and avoid blindly applying PA Guidelines (43, 44). In interviews, experienced physiotherapists suggest that promoting the PA Guidelines may be more suitable for short-term patients in outpatient settings. This viewpoint is also supported by a systematic review that indicates clinic-based, physiotherapist-led interventions in outpatient settings effectively increase PA levels in adults at risk of chronic diseases (45). In hospitals, outpatient departments have the highest patient flow (46), and they provide patients with opportunities to consult directly with physiotherapists about related issues (47). “Brief counseling,” the most commonly used method for physiotherapists promoting PA, makes it possible to intervene in PA during short interactions (48, 49). Such brief counseling and interventions mainly target suboptimal health conditions caused by insufficient PA, such as obesity and other chronic diseases (50). Extensive research has confirmed the benefits of PA in promoting health and preventing chronic diseases (51, 52). The PA Guidelines promotion may also be more effective and safer for these patients. Therefore, outpatient clinics may appear to be the optimal healthcare environment for physiotherapists promoting PA Guidelines.

## Strengths and limitations

This study represents the first of its kind targeting the rehabilitation student population in China, providing valuable empirical data to inform the teaching and application of PA guidelines in Chinese rehabilitation education. Notably, a mixed-methods approach was employed, which, in addition to quantitative data, included in-depth interviews with experienced rehabilitation therapists to explore the specific application of PA guidelines in clinical settings. It is essential to clearly differentiate between objective descriptions of results (observations) and interpretations based on those results (53). In the discussion, this study strives to maintain such a distinction and consciously integrates quantitative (“what”) and qualitative (“why” and “how”) findings to offer a more comprehensive and nuanced understanding. This integration provides empirical support for improving the instruction of PA guidelines in undergraduate rehabilitation education in China.

Several limitations must be acknowledged, primarily affecting generalizability. First, the quantitative sample used convenience sampling with a relatively low response rate, which may limit its representativeness of all Chinese rehabilitation students. Second, despite including students from different institutions, the sample had a higher proportion from the researchers' own university and did not cover a wider range, which may further affect representativeness. Finally, due to the limited sample size of the qualitative interviews (*n* = 10), the generalizability of the findings is constrained. Caution is warranted when extrapolating the results to inform broader curriculum or clinical-level recommendations. Most critically, the cross-sectional design of this study means that the observed associations cannot be interpreted as causal relationships. The direction of influence between education and reported practice behaviors requires verification through longitudinal or interventional studies. From a methodological standpoint, two additional aspects of the study could be further strengthened. First, all behavioral data were collected through self-report measures, which may be subject to certain subjective influences. Second, the survey instruments employed in this study have not yet undergone standardized validation, and thus, the precision of the measurements could be enhanced in future work.

Given this, the study suggests that future research should investigate the knowledge and practice of PA guidelines among a broader range of healthcare professionals, with a focus on bridging the gap between guideline knowledge and clinical practice. Building on this, priority could be given to optimizing the education and training of rehabilitation students. For instance, integrating PA guideline instruction more closely with clinical internships, particularly outpatient internships, could be considered to enhance their ability to apply PA guidelines in physical therapy practice, thereby promoting patient health.

## Conclusions

This study suggested that most Chinese rehabilitation students do not receive adequate teaching of PA Guidelines during their undergraduate curriculum and have a relatively low level of knowledge of the WHO's PA Guidelines. To remedy this deficiency, it is necessary to include health science-related courses in the undergraduate curriculum of rehabilitation students and integrate the teaching of the PA Guidelines with practical application. This study also brings attention to the fact that not all healthcare settings are equally suitable for promoting PA Guidelines. As a result, outpatient promotion may be more appropriate for short contact between physiotherapy and patients.

## Data Availability

The original contributions presented in the study are included in the article/Supplementary material, further inquiries can be directed to the corresponding author.
