# Supplementary material for: Mixed method assesses Chinese rehabilitation students' knowledge, attitude, and practice of physical activity guidelines
Source: Front Public Health. 2026 Apr 8;14:1775288. doi: 10.3389/fpubh.2026.1775288 (PMC13099770; doi:10.3389/fpubh.2026.1775288)
Supplement: Supplementary file 1 [file Data_Sheet_1.pdf]

Appendix 1: Online questionnaire design

筛选 screening

1-Please confirm that you are studying rehabilitation and related disciplines in China.
请确认您在中国且学习康复以及相关专业。

2-Which university do you belong to? 您属于哪个大学

XX university

3-Which degree do you belong to? 您属于哪个学位?

Options: Rehabilitation therapy/ Sports rehabilitation/ Others

选项：康复治疗学/技术 运动康复学/技术

4-Year of graduation 毕业年份

Options: 2020, 2021, 2022, 2023, other automatic exits

选项：2020，2021，2022，2023, 其他自动退出

5.- Which medical institution do you practice or work in? 您在哪个医疗机构实习或工作?

Options: public/private hospital, primary care, secondary, community, other

选项： 公立/私立医院，初级保健，二级保健，社区，其他

6. Approximately how many years of rehabilitation work or internship experience do you have? **您有大约多少年的康复工作或者实习经验?**

Options: 1 year, 2 years, 3 years, 4 years, or other

选项： 1 年，2 年，3 年，4 年，其他

7. Have you been taught physical activity guidelines during your university studies **您在大学学习过程中有被教授过身体活动指南吗?**

Yes, no. **是，不是,**

8. Have you ever heard of physical activity guidelines during your internship or work experience? **您在实习或者工作过程中有听说过身体活动指南吗?**

Yes, no. **是，不是,**

9. Do you know the recommended number of minutes of moderate-intensity physical activity per week for adults? Please indicate in numerical terms. **您是否知道建议成年人每周进行多少分钟的中等强度体育活动？请用数字表示。**

10. Do you know how many minutes of vigorous physical activity are recommended for adults per week? Please indicate with a number. **您知道建议成年人每周进行多少分钟的剧烈运动吗？请用数字表示。**

11. Do you know how many days per week of strength training is recommended for adults? Please use numbers only. **您知道建议成人每周做多少天的力量训练吗？请只使用数字**

12. Do you initiate conversations with rehabilitation clients about physical inactivity when on the internship or during work? **在实习或者工作期间，您是否会主动与康复对象进行有关身体不活动的谈话?**

Options: Never, Sometimes, Usually, Always.

选项： 从不，有时，通常，总是。

13. Do you supervise physical activity outside of therapy in your internship or at work? **在实习或者工作中您是否会在治疗以外督促患者进行身体活动?**

Options: Never, Sometimes, Usually, Always.

选项： 从不，有时，通常，总是。

14. Do you provide brief interventions for physical inactivity when required for treatment? **当治疗需要时，您是否提供针对身体不运动的简短干预措施?**

Options: Never, Sometimes, Usually, Always.

选项：从不、有时、通常、总是。

15. When you first see a new patient at the hospital where you work or intern, does your hospital formally assess whether the client falls into the risk category of physical inactivity (i.e. do you use any screening tools)? **当您所在的医疗机**

构初次接触病人时，您是否会正式评估服务对象是否属于缺乏体育锻炼的风险类别（即您是否使用任何筛选工具）？

Options: Never, Sometimes, Usually, Always.

选项： 从不，有时，通常，总是。

16. When promoting physical activity (PA) guidelines in hospitals, please choose the 2 methods you agree with most from the following **在医院里推广身体活动指南时，请从下列方法中选择您最赞同的 2 种方法**

A. To promote PA guidelines in hospitals, we can emphasize how important it is for preventing, treating, and improving health conditions. By doing this, we can encourage people to adopt positive physical activity behaviors.

A. 为了在医院推广身体活动指南，我们可以强调它对预防、治疗和改善健康状况是多么重要。通过这样做，我们可以鼓励人们采取积极的身体活动行为。

B. To promote PA guidelines in hospitals, we can help patients feel more willing and motivated by boosting their confidence and giving them the right support and guidance. That way, they can feel more successful in achieving their goals.

B. 为了在医院推广身体活动指南，我们可以通过增强病人的信心，给予他们正确的支持和指导，帮助他们感到更愿意和有动力。这样，他们就能感觉到更成功地实现他们的目标。

C. To promote PA guidelines in hospitals, we can show patients how to do different exercises and encourage them with positive feedback. This can help them feel more comfortable and motivated to keep moving.

C. 为了在医院推广身体活动指南，我们可以向病人展示如何做不同的练习，并用积极的反馈来鼓励他们。这可以帮助他们感到更舒适，并有动力继续运动。

D. To promote PA guidelines in hospitals, we can give them information and support them in finding the kind of physical activity they enjoy most. By doing this, we can help them feel more in control and motivated to keep up with their activity.

D. 为了在医院推广身体活动指南，我们可以给他们提供信息，支持他们找到他们最喜欢的体育活动。通过这样做，我们可以帮助他们感到更有控制力，并有动力保持活动。

Appendix 2. Semi-structured interview design

Hello, I'm Manyue Luo and I'm from the University of Edinburgh, thank you very much for taking part in my interview and I'm delighted to be able to talk to you today. Please tell us a bit about your experience with physical activity guidelines during your rehabilitation internship or work phase.

-What was your undergraduate degree in?
-How long have you had your internship or work experience?

1. Do you feel that physical activity is important in physiotherapy?

a - Yes, what is the important role of physical activity in physiotherapy? Can you give me an example?

b- No, do you understand the benefits of physical activity?

Describe the benefits of physical activity for participants.

Improving cardiovascular health: exercise can improve heart function and blood circulation, lower blood pressure and cholesterol levels and prevent cardiovascular disease.

Weight management: Exercise can help burn calories, control weight, and reduce body fat.

Improves physical fitness: Exercise can enhance muscle strength, endurance, and flexibility, improving physical fitness and preventing sports injuries.

Promotes mental health: Exercise can reduce anxiety, depression, and stress, and improve self-esteem and self-confidence.

Boosts immunity: Proper physical activity enhances the function of the immune system and prevents infection and disease.

Delays aging: Exercise improves the body's metabolism and cell regeneration, slowing down the body's aging process.

2. Are you aware of the physical activity guidelines?

a- Yes, please describe what they are and why they are important in a healthcare setting.

b- No, describe the physical activity guidelines

The physical activity guidelines are a guide to the amount and type of physical activity that is needed to help different groups of people (e.g. adults, children, older people, pregnant women, and people with disabilities) adopt a healthier lifestyle and reduce the negative effects of inactivity. According to the guidelines, adults need to perform at least 150 minutes of aerobic and muscle-strengthening exercise per week. For muscle-strengthening training, the guidelines also make specific recommendations, such as the need to perform at least two or more sessions per week, each of which should include different muscle groups such as the back, legs, buttocks, and arms. Each muscle group should be trained between 8-12 times and there should be sufficient rest between each movement. These guidelines are designed to encourage everyone to exercise more and live healthier!

So do you now have a general idea of the PA guidelines? It is the WHO's optimal dose of activity for each different group of people, as summarized by many authoritative academic and experimental studies.

3. Do you think this physical activity guideline is important in a healthcare setting? Why?

4. Did you study the physical activity guidelines at the university level including internship?

a- Yes, was the knowledge you learned about the guidelines well applied in practice, and in what main areas could they be applied?

b- No. What was the main content of the physiotherapy studies at the university level? What would be the main focus of knowledge regarding muscle strengthening?

5. At which stage do you think it would be better to include the teaching of physical activity guidelines throughout the rehabilitation studies? Would it be better to teach the basics or the practical phase? Or would it be better to have instruction at both stages?

6. If the Physical Activity Guidelines were to be taught at a later stage of the placement, how do you think it would be more helpful to promote the Physical Activity Guidelines? What are some ways in which physical activity guidelines can be taught in a healthcare setting?

7. Do you assess the past physical activity level of new patients at the time of first contact with them during the internship or work?

a - Yes, what are the main areas that are assessed? Would the results of this assessment be useful in setting up a rehabilitation program later on?

b- No. Do you think it is important to know the patient's past physical activity level? If so, would it help set up a rehabilitation program later?

8. In a general rehabilitation program, how many muscle-strengthening exercises are there per week? Is it at least three times a week? Is there a set schedule of training and rest times per week for the patient? Will it be adjusted according to

the patient's overall condition? How is it adjusted?

9. Apart from daily rehabilitation, are there any routines that deliberately encourage physical activity, or are there plans to help the patient develop good exercise habits?

a- Yes, have the patients you know continued to be followed up after discharge from the hospital? Are they still able to maintain a good level of physical activity?

b- No, have the patients you know been followed up after discharge? Are they still able to maintain a good level of physical activity? (compare)

10. Based on your experience in practice or at work, do you agree that among patients with similar levels of functional impairment, those who had an appropriate level of physical activity before their illness have a better chance of recovery than less active patients?

a- Yes, please tell us your opinion

b-Disagree, please tell us your opinion

11. What are your plans or ideas for the future application of the physical activity guidelines in rehabilitation practice? Do you have any suggestions or ideas on how to promote this PA guide in hospitals?

半结构访谈设计

您好，我是罗曼月，我来自爱丁堡大学，非常感谢您参加我的采访，今天我很高兴能采访您。请告诉我们关于您在康复实习和工作阶段对于身体活动指南方面的一些信息吧。

-您本科的专业是?

-您大概已经多长时间的实习或者工作经验?

1.您觉得身体活动在物理治疗中重要吗?

a-重要，身体活动在物理治疗中起到什么重要作用？可以向我举个例子吗

b-不重要，那您了解身体活动的好处吗?

介绍身体活动对病人的好处。

改善心血管健康：运动可以提高心脏功能和血液循环，降低血压和胆固醇水平，预防心血管疾病。

控制体重：运动可以帮助燃烧卡路里，控制体重，减少体脂肪。

提高身体素质：运动可以增强肌肉力量、耐力和灵活性，提高身体素质，预防运动损伤。

促进心理健康：运动可以减轻焦虑、抑郁和压力，提高自尊心和自信心。

提高免疫力：适当的身体活动可以增强免疫系统的功能，预防感染和疾病。

延缓衰老：运动可以提高身体的新陈代谢和细胞再生能力，延缓身体的衰老过程。

2.您是否了解身体活动指南?

a-了解，请描述一下它们是什么，以及它们为什么对医疗环境很重要

b-不了解，介绍身体活动指南 身体活动指南是一份关于身体活动量和类型的指南，旨在帮助不同人群（比如成人、儿童、老年人、孕妇和残疾人）采取更健康的生活方式，减少因为不活动带来的消极影响。根据指南，成年人每周至少需要进行 150 分钟的有氧运动和肌肉强度训练。对于肌肉强化训练，指南还特别提出了一些建议，比如每周至少需要进行两次以上的训练，每次训练应该包括不同的肌肉组群，如背部、腿部、臀部和手臂等。每个肌肉组群的训练次数应该在 8-12 次之间，每个动作之间应该有充分的休息时间。这些指南旨在鼓励大家多运动，健康生活！

那您现在对身体活动指南是否有了一个大概的了解？其实它就是 WHO 世界卫生组织通过许多权威的学术和实验总结出的对每个不同人群的最佳活动剂量。

3.您觉得这个身体活动指南在医疗环境中重要吗？为什么？

4.在大学阶段包括实习有没有学习过身体活动指南？

a-有，学习的关于指南的知识在实践中有没有得到很好应用，主要在哪些方面可以应用？

b-没有，大学阶段学习物理治疗学的主要内容涉及什么？关于肌肉强化方面会重点掌握哪些知识？

5.您觉得在整个康复学习阶段，哪个阶段加入身体活动指南教学会比较好？是基础知识教学还是实习阶段？或者在两个阶段都有教学会更好？

6.如果在以后的实习阶段加入身体活动指南的教学，您觉得如何教学会对推广身体活动指南更加有帮助？有哪些方法可以将身体活动指南教学应用于医疗环

境中?

7.在实习或者工作阶段，首次接触新病人时是否会对其过去身体活动水平进行评估?

a-有，主要会从哪些方面评估？这个评估结果对以后设置康复计划有没有帮助呢?

b-没有，您觉得了解病人过去的身体活动状况重要吗？如果评估的话对之后制定康复计划有没有帮助呢?

8.在一般的康复计划中，每周有多少次肌肉强化练习？是否每周至少有三次？患者每周训练和休息的时间有没有固定的安排？会不会根据患者的整体情况来调整？如何调整

9.除了日常的康复训练外，是否有刻意鼓励身体活动的常规训练或有在计划帮助病人养成良好的锻炼习惯?

a-有，您所知道的病人在出院后有没有继续追踪呢？他们还可以保持较好的身体活动水平吗?

b-没有，您所知道的病人在出院后有没有继续追踪呢？他们还可以保持较好的身体活动水平吗？（对比）

10.根据您实习或者工作中的经验来看，您是否认同在功能障碍程度相似的病人中，那些在患病前就有比较好的身体活动水平的病人是否比不太活跃的病人有

更好的康复机会?

a-认同，请谈谈您的看法（身体素质，运动习惯帮助训练）

b-不认同，请谈谈您的看法

11.您对未来在康复实践中身体活动指南的应用有什么计划或想法？如何在医院推广这个身体活动指南，有没有什么建议和想法?

Appendix 3

A list of all colleges and universities and corresponding numbers of rehabilitation students in the questionnaire.

Colleges and universities	Number of responses
Shandong First Medical University	61
Beijing Sport University	11
Henan University of Chinese Medicine	3
Tsinghua University	2
Nanjing University of Chinese Medicine	2
Guangzhou University of Chinese Medicine	2
Nanjing Sports Institute	2
Beijing City University	2
Sichuan Health Rehabilitation Vocational College	1
Shandong Sports Institute	1
Binzhou Medical College	1
Hunan University of Chinese Medicine	1
Shanghai University of Chinese Medicine	1
North China University of Science and Technology	1
Tianjin University of Chinese Medicine	1
Jining Medical College	1
Shandong Rehabilitation University	1
Gansu University of Chinese Medicine	1
Shanghai Sports Institute	1
Xuzhou Medical University	1
Shaoxing College of Arts and Sciences	1
Guangzhou Sports Institute	1
Tianjin Sports Institute	1
Qilu Medicine College	1
Southwestern University of Finance and Economics	1
Peking University	1
Harbin Medical University	1
Nanjing Medical University	1
**In total**	**105**
